# Prolonged hypothyroidism severely reduces ovarian follicular reserve in adult rats

**DOI:** 10.1186/s13048-017-0314-7

**Published:** 2017-03-16

**Authors:** Li Meng, Eddy Rijntjes, Hans J. M. Swarts, Jaap Keijer, Katja J. Teerds

**Affiliations:** 1 Human and Animal Physiology, Wageningen University, P.O. Box 338, 6700 AH Wageningen, The Netherlands; 2 College of Animal Science, South China Agricultural University, Guangzhou, 510642 People’s Republic of China; 3 Institut für Experimentelle Endokrinologie, Charité Universitätsmedizin Berlin, Augustenburger Platz 1, 13353 Berlin, Germany

**Keywords:** Ovary, Hypothyroidism, Growing follicle population, Primordial follicle reserve, Follicular atresia, AMH

## Abstract

**Background:**

There is substantial evidence both in humans and in animals that a prolonged reduction in plasma thyroid hormone concentration leads to reproductive problems, including disturbed folliculogenesis, impaired ovulation and fertilization rates, miscarriage and pregnancy complications. The objective of the present study is to examine the consequences of chronic hypothyroidism, induced in adulthood, for the size of the ovarian follicle pool. In order to investigate this, adult female rats were provided either a control or an iodide deficient diet in combination with perchlorate supplementation to inhibit iodide uptake by the thyroid. Sixteen weeks later animals were sacrificed. Blood was collected for hormone analyses and ovaries were evaluated histologically.

**Results:**

At the time of sacrifice, plasma thyroid-stimulating hormone concentrations were 20- to 40-fold increased, thyroxine concentrations were negligible while tri-iothyronin concentrations were decreased by 40% in the hypothyroid group, confirming that the animals were hypothyroid. Primordial, primary and preantral follicle numbers were significantly lower in the hypothyroid ovaries compared to the euthyroid controls, while a downward trend in antral follicle and corpora lutea numbers was observed. Surprisingly the percentage of atretic follicles was not significantly different between the two groups, suggesting that the reduced preantral and antral follicle numbers were presumably not the consequence of increased degeneration of these follicle types in the hypothyroid group. Plasma anti-Müllerian hormone (AMH) levels showed a significant correlation with the growing follicle population represented by the total ovarian number of primary, preantral and antral follicles, suggesting that also under hypothyroid conditions AMH can serve as a surrogate marker to assess the growing ovarian follicle population.

**Conclusions:**

The induction of a chronic hypothyroid condition in adult female rats negatively affects the ovarian follicular reserve and the size of the growing follicle population, which may impact fertility.

## Background

A disturbed thyroid status and concomitant alterations in thyroid hormone (TH) production are known to affect key functions in growth, development and metabolism, such as basal metabolic rate, energy production, and carbohydrate and lipid metabolism [1, 2]. Besides, there is substantial evidence that a prolonged reduction in TH concentration leads to a broad spectrum of reproductive problems, including disturbed folliculogenesis, impaired ovulation and fertilization rate, and in severe cases to complete ovarian failure [3–6].

In humans the majority of cases of hypothyroidism find their origin either in thyroid autoimmune disease (TAD), a condition affecting 5–20% of women of child bearing age, or insufficient iodine intake [7–10]. Not only in underdeveloped countries, but also in 34% of the developed countries, such as USA, UK, Italy, France and Ireland, iodine intake, especially in pregnant women, is insufficient [8, 11]. In the UK for instance, two thirds of pregnant women are iodine deficient and therefore at risk to develop hypothyroidism. This implies that hypothyroidism is a less rare condition in women at reproductive age than anticipated.

Despite it is generally acknowledged that TH disorders are associated with disturbed ovarian folliculogenesis, little is known about the precise effects of chronic hypothyroidism on follicular development and the ovarian follicular reserve. The number of animal studies investigating the effect of hypothyroidism on follicular development in adulthood is limited and the observations contradictory. For example, Ortega and colleagues [12] have reported that hypothyroidism in adult female rats induces a significant suppression in basal LH release, leading to ovarian atrophy. Hapon et al. [13] have pointed out that virgin hypothyroid rats show prolonged periods of vaginal dioestrus. In line with this Mattheij and colleagues [14] observed that in hypothyroid rats the oestrous cycle has become irregular, but if these females do present vaginal proestrous, the subsequent LH surge is much higher than in the euthyroid controls. Other investigators have reported small changes in LH and FSH release and the presence of mature follicles and corpora lutea [15], although pregnancy incidence and litter size were reduced under hypothyroid conditions when compared to euthyroid controls [16]. As far as we are aware a detailed analysis of the consequences of a prolonged exposure to reduced TH concentrations for the ovarian follicular reserve has not been performed yet.

In recent years, we have developed an animal model of diet-induced hypothyroidism by depleting the endogenous iodine stores of rats to study the effects of chronic hypothyroidism on ovarian follicular development [17]. In the present study we aim to use this animal model to investigate the effects of adult-induced chronic hypothyroidism on the size of the ovarian follicle pool, as depicted by the total number of primordial, primary, preantral and antral follicles per ovary. This will give us insight in the effects of adult hypothyroidism on ovarian follicular development and thus reproductive potential in rodents. The results of the present study may further contribute to the understanding of the consequences of hypothyroidism on the ovarian follicle population in women of reproductive age.

## Methods

### Chemicals

All chemicals were purchased from Sigma (Zwijndrecht, the Netherlands) unless indicated otherwise.

### Animals and treatment

Twenty 10-week-old female Wistar rats (HsdCpbWU) were obtained from Harlan (Horst, the Netherlands). The rats were housed individually and kept under controlled conditions (room temperature 20.5–21.5 °C; humidity 55–65%; light regimen 60–80 lux, lights on from 03:00 to 17:00 local daylight saving time). The animals had free access to food and tap water and were provided with cage enrichment in the form of a 10 cm sisal rope. Two weeks after arrival the female rats of the experimental group (*n* = 9) were put on an iodide-poor diet based on AIN 1993 requirements (Research Diet Services, Wijk bij Duurstede, the Netherlands) [18] supplemented with 0.75% sodium perchlorate in the drinking water to deplete endogenous iodie store [19, 20]. This treatment leads already within two weeks time to a 16-fold increase in plasma TSH concentrations [17]. The control group (*n* = 11) was at the same time put on normal drinking water without sodium perchlorate and was given a euthyroid control diet, consisting of the iodide-poor diet, supplemented with 7 μg iodide per 100 g dry weight of the diet to fulfill the normal iodide requirements of rats. The experimental hypothyroid diet was continued for 16 weeks, after which the animals were sacrificed. Rats were anesthetized using carbon dioxide and oxygen (flow: 1:2). Blood was obtained by heart puncture and collected in heparin-coated tubes. Rats were killed by decapitation and ovaries were dissected, fixed in Bouin’s fluid for 24–48 h and stored in ethanol 70% until further processing. Plasma was stored at −20 °C until further analysis. All animals were killed at the proestrous stage of the estrous cycle between 11.00 and 14.00 h. The stage of the oestrous cycle was determined by daily analysis of vaginal smears, starting approximately two weeks before sacrifice.

### Histological evaluation of ovarian tissues

The ovaries of 4 animals were embedded in paraffin and serially sectioned at a thickness of 5 μm. Every fifth section of each ovary was mounted on glass slides, stained with periodic acid Schiff’s reagent (PAS) and Mayer’s haematoxylin (Klinipath, Duiven, the Netherlands), and examined by light microscopy. From these sections the numbers of healthy primordial, primary, preantral and antral follicles were counted as described previously [17, 21, 22]. Briefly, follicles were scored as primordial if they contained an intact oocyte with a clear nucleus and nucleoli surrounded by a single layer of squamous pregranulosa cells. Follicles were scored as primary when they contained an intact, enlarged oocyte with a clear nucleus and nucleolus surrounded by a single layer of granulosa cells of which 50–100% had a cuboidal appearance. The granulosa layer of preantral follicles consisted of more than one layer of cuboidal cells, an oocyte with a clear nucleus and nucleolus and a developing theca layer. Antral follicles consisted of several layers of granulosa cells, an oocyte with a clear nucleus and nucleolus, an antrum of which the diameter was at least the size of the diameter of the oocyte, and a theca layer. In order to estimate the total number of follicles within one ovary, the number of primordial, primary, preantral and antral follicles counted in the mounted sections was multiplied by five to account for the fact that every fifth section was used in the follicle counting [17, 22].

Atretic preantral follicles were characterized by the presence of a degenerating oocyte, disorganized granulosa cell layer with the presence of a limited number of apoptotic nuclei, while the surrounding theca cells showed signs of hypertrophy. Antral follicles were considered to be atretic when more than 5% of the granulosa cells showed signs of apoptosis, while the theca layer of these follicles showed signs of hypertrophy. As atresia proceeded, the granulosa cells were lost completely and the oocyte degenerated, leaving remnants of the zona pellucida and hypertrophied theca cells. In order to prevent double counting of atretic follicles, we analysed in three sections of each ovary (at a quarter, half and three-quarters of the ovary) all preantral and antral healthy and atretic follicles, independently of the presence of an oocyte, as described previously with minor modifications [22, 23]. Since the counted numbers reflect only part of the total follicle population in an ovary, the mean number of atretic follicles was expressed as percentage of the number of non-atretic plus atretic follicles. Primordial and primary follicles were excluded from this counting procedure [17, 23].

In order to determine the number of corpora lutea (CLs) per ovary, overview pictures were taken of every 20^th^ ovarian section using a Zeiss Axioscoop II microscope equipped with a MRc5 camera and Axiovision 4.8.0.0 software (Zeiss GmbH, Jena, Germany). By following the CLs throughout the ovary it was possible to determine the total number of CLs per ovary.

### Radio immunoassays

Total thyroxine (T_4_) (DSL-3200; DSL, Webster, TX, USA) and total triiodothyronine (T_3_) (DSL-3100) concentrations were assayed according to the manufactures protocol. Luteinizing hormone (LH), follicle stimulating hormone (FSH) and TSH concentrations were determined by validated in-house double-antibody RIAs for rat serum analysis [14, 24, 25] using materials supplied by the National Institute of Diabetes, Digestive and Kidney Diseases (NIDDK; Bethesda, MD, USA). For all in-house RIAs SACcel (donkey anti-rabbit) was used as the secondary antibody. The levels of the different hormones were expressed in terms of NIDDK standards. The detection limits for the assays were: 5 ng/ml for total T_4_, 0.25 ng/ml for total T_3_, 0.03 ng/ml for LH, 0.1 ng/ml for TSH and 0.4 ng/ml for FSH. The intra- and inter-assay variation was determined using several pools of rat serum and was less than 11% for all purchased RIAs and less than 9.5% for all in-house RIAs.

### Plasma anti-Müllerian hormone ELISA

Plasma anti-Müllerian hormone (AMH) concentrations were analysed using the AMH Gen II ELISA assay according to the manufacturers instructions (Beckman Coulter, Sinsheim, Germany). The detection limit of the assay was 0.16 ng/ml; the intra-assay coefficient of variation was 5.3%.

### Statistical analysis

Data were expressed as mean ± standard error of the mean (SEM). GraphPad Prism version 5.03 (Graphpad Software, San Diego, USA) was used for statistical analysis. Data were checked for normality and when normality was confirmed the Student’s *t* test was used for data analysis. In case normality could not be assumed, data were log10 transformed. *P*-values < 0.05 were considered significantly different.

## Results

### Thyroid status

**Fig. 1:**
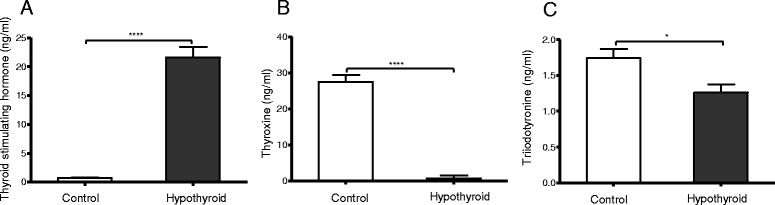
Plasma concentrations of thyroid-stimulating hormone (TSH) (**a**), thyroxine (T_4_) (**b**), and tri-iodothyronine (T_3_) (**c**) were measured in euthyroid control rats (open bars, *n* = 11) and hypothyroid rats (filled bars, *n* = 9). Values represent mean ± SEM. ****p < 0.0001; *p < 0.05

To assess the thyroid status of the rats, plasma TSH, T_4_ and T_3_ concentrations were determined. Within 2 weeks after the start of the dietary intervention plasma TSH concentrations had increased by approximately 15-fold in the hypothyroid rats. By 16 weeks, TSH concentrations were even further increased to approximately 24-fold the concentrations in the age-matched controls (Fig. 1a,). TSH concentration in control animals ranged from 0.34 to 1.52 ng/ml, while in hypothyroid animals from 15.86 to 34.04 ng/ml. Concomitantly, T_4_ concentrations in the hypothyroid animals had become barely detectable (Fig. 1b), while plasma T_3_ levels were reduced by approximately 30%. (Figure 1c). Plasma T_4_ and T_3_ concentrations in control animals ranged from 20.30 to 39.1 ng/m and 1.08 to 2.26 respectively, and in hypothyroid females from not detectable to 5.6 g/ml and 0.81 to 1.62 respectively.

### Plasma FSH, LH and AMH concentrations

**Fig. 2:**
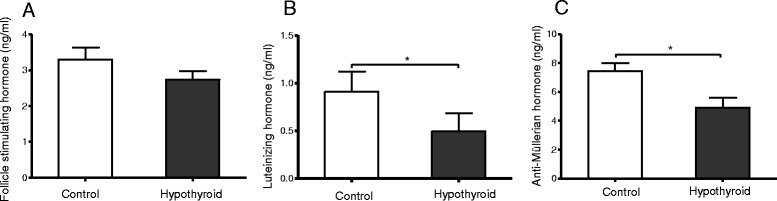
Plasma follicle-stimulating hormone (FSH) (**a**), luteinizing hormone (LH) (**b**) and anti-Müllerian hormone (AMH) concentrations (**c**) in euthyroid control rats (open bars, *n* = 11) and hypothyroid rats (filled bars, *n* = 9). Values represent mean ± SEM. *p < 0.05

No difference was detected in plasma FSH concentrations between the hypothyroid female rats and the age-matched control rats (Fig. 2a). Plasma LH and AMH concentrations, however, were approximately 40 and 30% lower, respectively, in the hypothyroid animals compared to the age-matched euthyroid controls (Fig. 2b and c).

### Follicular development

**Fig. 3:**
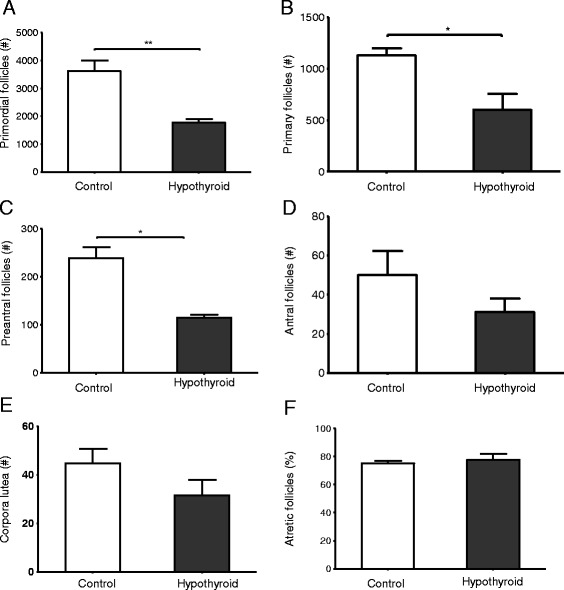
Effects of mild hypothyroidism on ovarian follicle numbers in euthyroid control rats (open bars) and hypothyroid rats (filled bars). Total number of ovarian primordial (**a**), primary (**b**), preantral (**c**), antral follicles (**d**), total ovarian number of corpora lutea (CLs) (**e**) and percentage of atretic follicles (**f**). Values represent mean ± SEM; *n* = 4. *p < 0.05; **p < 0.01

In order to obtain further insight in the effects of prolonged hypothyroidism on ovarian follicle reserve in adult rats, the total number of follicles per ovary was determined. After 16 weeks of hypothyroidism the primordial follicle number was approximately 60% lower in the hypothyroid females compared with the age-matched euthyroid controls (Fig. 3a). The number of primary (Fig. 3b) and preantral (Fig. 3c) follicles had decreased by about 40% in the hypothyroid rats compared with the controls, while a downward trend was observed in antral follicle number, though this did not reach the level of significance (Fig. 3d). In line with these observations the total number of CLs per ovary showed a downward trend in the hypothyroid females compared to the euthyroid controls, though this did not reach the level of significance (Fig. 3e).

To investigate whether the observed decrease in follicle numbers was due to follicular degeneration, the percentage of atretic follicles was determined. The results showed that there was no significant difference in the percentage of atretic follicles between hypothyroid rats and age-matched controls (Fig. 3f).

**Fig. 4:**
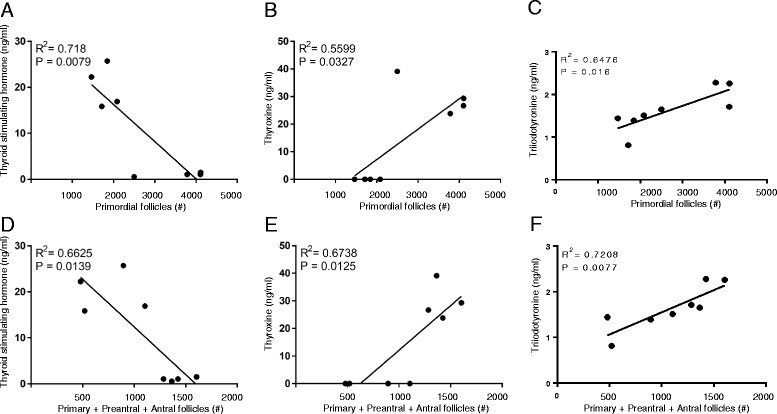
Correlations between the ovarian follicle numbers and thyroid status: primordial follicle number and TSH concentration (**a**), T_4_ concentration (**b**) and T_3_ concentration (**c**) respectively; primary + preantral + antral follicle number and TSH concentration (**d**), T_4_ concentration (**e**) and T_3_ concentration (**f**) respectively

Correlation analysis showed that there existed a significant correlation between THS levels and primordial follicle numbers in both control and hypothyroid females, as well as between T_4_ and T_3_ levels and primary + preantral + antral follicle numbers, the growing follicle population (Fig. 4).

**Fig. 5:**
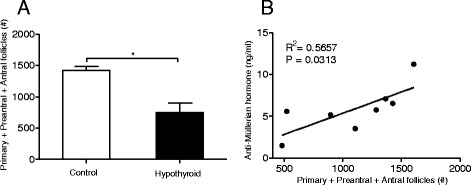
Effects of mild hypothyroidism on the size of the growing ovarian follicle population (consisting of primary plus preantral and antral follicle numbers) in euthyroid control (open bar) and hypothyroid rats (filled bar) (**a**), and the correlation between the growing ovarian follicle population and plasma AMH concentration in hypothyroid rats (**b**). Values represent mean ± SEM; *n* = 4

There is some dispute about the use of plasma AMH concentrations as marker of ovarian follicular reserve [26–28]. According to Findlay and colleagues [27] AMH should be related to the growing follicle population (the ovulatory potential), excluding the primordial follicle pool. We therefore performed a correlation analysis between plasma AMH concentrations and the growing follicle population and observed a significant correlation between AMH and the growing follicle population (Fig. 5). No correlation was observed between plasma AMH concentrations and primordial follicle numbers (data not shown).

## Discussion

The present study is, to our knowledge, the first that provides a detailed analysis of the follicular reserve in adult female rats that are exposed to chronic hypothyroid conditions for a prolonged period of time. Primordial, primary and preantral follicle numbers are significantly lower in the hypothyroid ovaries compared to the euthyroid controls, while a downward trend in antral follicle numbers and corpora lutea is observed. Quite surprisingly the percentage of atretic follicles was not different between the two groups, suggesting that the reduced preantral and antral follicle numbers in the hypothyroid group are not likely due to increased degeneration of these follicle types.

The reduction in the number of primordial follicles in the hypothyroid rats suggests that either recruitment of primordial follicles into the pool of growing follicles is enhanced or alternatively the reduced circulating TH concentrations induce increased primordial follicle degeneration. Assuming that the initial recruitment of primordial follicles is indeed enhanced, this should lead to an increase in the primary follicle pool size. Our data do not support this assumption; on the contrary, primary follicle numbers are decreased significantly as are preantral follicle numbers. This implicates that recruited primordial and/or primary follicles may have degenerated before reaching the preantral stage. The manifestation of attrition in primordial and primary follicles is thought to be of short duration [29, 30] and therefore difficult to identify by plain histology. We may have simply missed these atretic follicles during our analysis. Furthermore, we have collected ovarian tissue at only one time-point, namely 16 weeks after the start of the dietary intervention. We therefore do not know whether degeneration of these follicles takes place gradually over time, or whether follicles degenerate massively during the initial period of the dietary intervention when TH concentrations are decreasing, followed by a new equilibrium between recruitment and degeneration when TH concentrations have stabilized. Based on the observation that the number of preantral follicles is reduced by approximately the same percentage as the primary follicle number, and antral follicle numbers and CL numbers not being different from euthyroid control values, we favour the first explanation, i.e. gradual depletion.

Plasma LH concentrations are significantly decreased in chronic adult hypothyroid rats compared with the euthyroid controls. This observation is in agreement with previous reports in female rats by the groups of Hatsuta [31] and Tohei [32]. This reduction in LH concentrations may be explained by an elevation in prolactin concentration, a phenomenon observed both in rats and humans [5, 31, 33]. Due to the disturbed thyroid function, thyrotropin-releasing hormone (TRH) release by the hypothalamus is increased, stimulating not only pituitary TSH but also prolactin synthesis and release [34]. Elevated prolactin concentrations exert a negative influence on the pituitary response to gonadotropin releasing hormone (GnRH) [31] leading to a lower LH secretion. Despite these reduced basal LH concentrations ovulation is not necessarily completely blocked under hypothyroid conditions [5, 14, 32]. On the contrary, the preovulatory LH peak may even be augmented, facilitating ovulation as has been shown previously [14].

The significant reduction in primordial and primary follicle numbers in the hypothyroid ovaries in the present study is in sharp contrast to our previous experiment where the hypothyroid condition was induced during foetal life and continued postnatally until sacrifice [17]. Here we did not notice a significant difference in primordial and primary follicle numbers between the hypothyroid and age-matched control rats. These seemingly contradictory observations suggest that the age at onset of the hypothyroid condition may determine the extend of the effect on the ovarian follicle pool. One has to keep in mind, however, that the switch from a euthyroid to a hypothyroid diet in adulthood leads to a 10 to 15-fold increase in plasma TSH levels within two weeks time, while in the study by Meng et al. [17] rats have been exposed to these reduced TH concentrations during their entire life. This distinction in time of onset of hypothyroidism may influence whole body metabolism and the ovarian micoenvironment differently, an assumption supported by the observed differential effects on plasma LH concentrations. Hence, the apparent discrepancy in effects of chronic hypothyroidism on the ovarian follicle pool may very well be explained by the time of onset of the hypothyroid condition.

Despite the fact that abnormalities in thyroid function have been associated with subfertility [5, 35], in most developed countries, national guidance does not recommend routine measurement of thyroid function in women with fertility problems [35, 36]. Our data, however, implicate that prolonged hypothyroidism negatively affects reproductive poptential. A decrease in size of the ovarian follicle pool including non-growing and growing follicles as observed in the present study, may be correlated with a decline in female fertility, stressing the importance of knowledge concerning the reproductive potential in women with fertility and thyroid problems. Numerous studies in rats and humans have reported that AMH can be used as a surrogate marker for ovarian follicular reserve (e.g. [26, 28, 37, 38]). Nevertheless, Findley and colleagues [27] recently questioned the use of biomarkers such as serum AMH as indicator of ovarion follicular reserve. According to these authors these biomarkers do not provide direct measurement of the size of the “resting” pool of primordial follicles, but only reflect the presence of growing follicles (in fact presumably mostly antral follicles). Our data are in line with this suggestion as plasma AHM concentrations under hypothyroid conditions correlate with the growing pool of follicles, also named ovulatory portential by Findley et al. [27], and do not correlate with the number of primordial follicles, the primordial ovarian reserve [27].

Several studies have investigated the relation between assisted reproductive technology (ART) outcomes, AMH levels and the presence of TAD in women. The results of these studies are variable. Polyzos et al. for example did not observe an association between thyroid disorders and TAD, and low ovarian reserve as estimated by AMH levels [39]. Magri et al. on the other hand found a negative influence of TAD on ART outcome in women with a normal ovarian reserve as depicted by high serum AMH levels [40]. Taking into consideration that one needs to be careful in extrapolating results from animal studies directly to the human condition, an explanation for the discrepancy among these studies may be that in the studies by Polyzos et al. [39] and Magri et al. [40] subclinical hypothyroidism prevailed while is the present study TSH concentrations were increased approximately 24-fold, indicative for a more severe disturbed thyroid function.

Taken together, the results of the present study show that prolonged mild hypothyroidism negatively influences the ovarian follicular reserve as well as the size of the growing follicle population, which may negatively impact fertility. Furthermore, also under hypothyroid conditions plasma AMH can be used as a biomarker of the ovarian population of growing follicles.

## Conclusions

The induction of chronic hypothyroidism in adult female rats may negatively impact fertility as it leads to a significant reduction in primordial, primary and preantral follicle numbers, and a downward trend in antral follicle and corpora lutea numbers. Whether the enhanced depletion of the ovarian reserve will also lead to a premature exhaustion of the ovarian follicle pool, or whether a new exquilibrium in follicle recruitment will be established, needs to be investigated.

## Abbreviations

AMH: Anti-Müllerian hormone
CLs: Corpora lutea
FSH: Follicle-stimulating hormone
GnRH: Gonadotropin-releasing hormone
LH: Luteinizing hormone
PAS: Periodic acid Schiff’s reagent
T_3_: Triiodothyronine
T_4_: Thyroxine
TH: Thyroid hormone
TRH: Thyrotropin-releasing hormone
TSH: Thyroid-stimulating hormone
